# Adult Protective Services and Law Enforcement: Does It Add or Subtract to the Precarity of Living Alone With Dementia?

**DOI:** 10.1093/geroni/igaa057.2025

**Published:** 2020-12-16

**Authors:** Michael Splaine

**Affiliations:** Splaine Consulting, Columbia, Maryland, United States

## Abstract

In 2014, more than 12.5 million people age 65+ lived alone in the U.S. Of these, approximately one third had a cognitive impairment. Although protective services may identify risks to such individuals, they may not have a full understanding of the notion of precarity, or the looming uncertainty regarding space and place, that solo dwellers experience. This presentation explores the tension between the intentions of protective services and the experience of precarity for persons living alone. More specifically, persons living alone with dementia participating in online groups and community events report feelings of risk of loss of autonomy and rights if their status becomes known. The presenter will review these impressions against current police and adult protective services policies and standard practices.

